# Activation and Regulation of the Pattern Recognition Receptors in Obesity-Induced Adipose Tissue Inflammation and Insulin Resistance

**DOI:** 10.3390/nu5093757

**Published:** 2013-09-23

**Authors:** Yasuharu Watanabe, Yoshinori Nagai, Kiyoshi Takatsu

**Affiliations:** 1 Department of Immunobiology and Pharmacological Genetics, Graduate School of Medicine and Pharmaceutical Science for Research, University of Toyama, 2630 Sugitani, Toyama-shi, Toyama 930-0194, Japan; E-Mail: yasuharu@med.u-toyama.ac.jp; 2 Toyama Prefectural Institute for Pharmaceutical Research, 17-1 Nakataikouyama, Imizu City, Toyama 939-0363, Japan

**Keywords:** adipose tissue inflammation, cytokine, inflammasome, insulin resistance, metabolic syndrome, obesity, pattern recognition receptor, TLR, type 2 diabetes mellitus

## Abstract

Obesity-associated chronic tissue inflammation is a key contributing factor to type 2 diabetes mellitus, and a number of studies have clearly demonstrated that the immune system and metabolism are highly integrated. Recent advances in deciphering the various immune cells and signaling networks that link the immune and metabolic systems have contributed to our understanding of the pathogenesis of obesity-associated inflammation. Other recent studies have suggested that pattern recognition receptors in the innate immune system recognize various kinds of endogenous and exogenous ligands, and have a crucial role in initiating or promoting obesity-associated chronic inflammation. Importantly, these mediators act on insulin target cells or on insulin-producing cells impairing insulin sensitivity and its secretion. Here, we discuss how various pattern recognition receptors in the immune system underlie the etiology of obesity-associated inflammation and insulin resistance, with a particular focus on the TLR (Toll-like receptor) family protein Radioprotective 105 (RP105)/myeloid differentiation protein-1 (MD-1).

## 1. Introduction

Obesity has become a worldwide health problem because it is strongly associated with metabolic syndromes including type 2 diabetes mellitus (T2DM), atherosclerosis and ischemic heart diseases [1,2]. Accumulating evidence indicates that chronic-low grade inflammation has a crucial role in the pathogenesis of obesity-related metabolic dysfunction [3,4]. The chronic inflammatory alternations are associated with dynamic changes in the composition and function of immune cells in various tissues such as adipose tissue, pancreatic islet, liver, muscle, and hypothalamus [5,6,7]. In addition, crosstalk among various cells in these tissues regulates chronic inflammation [8].

**Figure 1:**
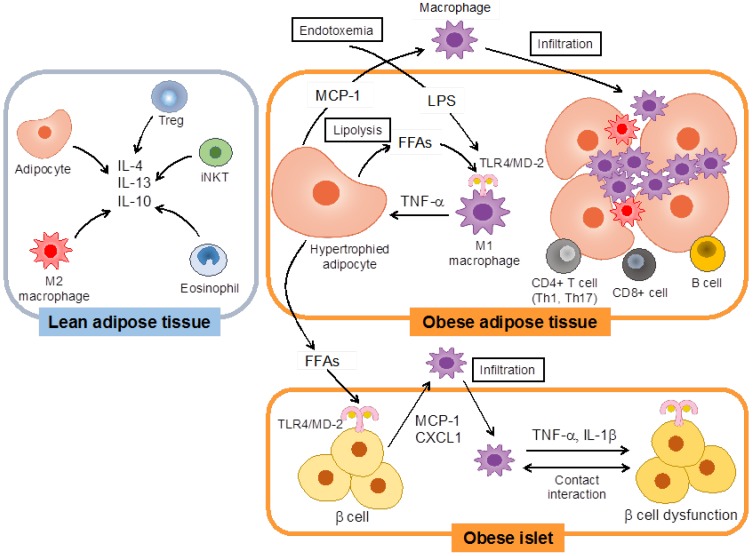
Inflammatory changes in adipose tissue and pancreatic islets in association with obesity. In the lean state, regulatory T (Treg) cells, esosinophils, invariant natural killer T (iNKT) cells, M2-like resident macrophages, and adipocytes secrete anti-inflammatory cytokines including interleukin (IL)-4, IL-13 and IL-10 and suppress inflammation in adipose tissue. However, in the obese state, macrophages are activated by recognition of free fatty acids (FFAs) from hypertrophied adipocytes or lipopolysaccharide (LPS) through the Toll-like receptor (TLR) 4/myeloid differentiation protein-2 (MD-2) complex to induce tumor necrosis factor-α (TNF-α) production. Adipocytes secrete monocyte chemotactic protein-1 (MCP-1) in response to TNF-α and promote filtration of macrophages into adipose tissues. In addition, CD4+ or CD8+ T cells and B cells infiltrate adipose tissue during obesity and exaggerate adipose tissue inflammation. FFAs such as palmitate from adipocytes may activate TLR4/MD-2 expressed in cells and induce the production of chemokines, chemokine (C-X-C motif) ligand 1 (CXCL1) and MCP-1. In response to these chemokines, macrophages infiltrate into islets. Cytokines such as TNF-α and IL-1β from macrophages and contact interactions between cells and macrophages lead to cell dysfunction.

Key to understanding the pathogenesis of adipose tissue inflammation is realization that large numbers of immune cells reside in adipose tissue [4]. In the lean state, regulatory T (Treg) cells [9], eosinophils [10], invariant natural killer T (iNKT) cells [11] and M2-like resident macrophages predominate in adipose tissue [12]. These cells secrete the anti-inflammatory cytokine interleukin (IL)-10 and T helper (Th) 2 cytokines that also suppress inflammation in adipose tissue (Figure 1) [13,14]. Adipocytes secrete adiponectin and Th2 cytokines, which maintain the homeostatic state of adipose tissue [15]. On the other hand, inflammatory immune cells infiltrate adipose tissue during the course of obesity. A large number of M1-like macrophages accumulate in adipose tissue and become major sources of inflammatory mediators such as tumor necrosis factor-α (TNF-α) and IL-6 (Figure 1) [16]. It has been reported that the infiltration of CD8+ T cells [17] and neutrophils precedes macrophage accumulation and promotes the recruitment and activation of macrophages [18]. However, the order of cell infiltration into adipose tissue and the sequence of events that lead to macrophage infiltration remain unclear. A balance of CD4+ T cell subsets is also critical for the regulation of adipose tissue inflammation (Figure 1). The number of interferon (IFN)-γ-secreting CD4+ T cells (Th1 cells) is increased in obese adipose tissue from mice and humans, whereas the number of Treg cells is decreased [9,19]. The number of IL-17-secreting CD4+ T cells (Th17 cells) is also increased in obese subcutaneous adipose tissue and blood in mice and humans [19,20,21]. Furthermore, Treg cells are decreased in the peripheral blood of patients with T2DM, whereas Th1 and Th17 cells are increased [22]. Intriguingly, CD4+ Th1 cells in visceral adipose tissue interact with antigen-presenting cells, such as adipose tissue macrophages (ATMs), and proliferate and produce IFN-γ *in vitro* and *in vivo* [23]. In addition, B cells also have been implicated in insulin resistance through production of pathogenic immunoglobulin G (IgG) antibodies and pro-inflammatory cytokines, which regulate Th17/Th1 cell functions and Treg cell population in obesity (Figure 1) [24,25]. Moreover, the levels of IL-10 are decreased in B cells from T2DM [26]. B cell-null mice have less high-fat diet (HFD)-induced insulin resistance [24]. These reports suggest that B cells also play an important role in diabetes.

Not only adipose tissue but also other organs can be affected by the chronic inflammation associated with metabolic syndrome. As in adipose tissue, macrophages accumulate in pancreatic islets with diet-induced obesity and produce pro-inflammatory cytokines [27]. Inflammation in islets triggers their apoptosis and reduces insulin secretion from cells, leading to decreased islet mass [28]. Secreted cytokines, such as TNF-α and IL-1β, can directly inhibit insulin secretion as well as insulin signaling, and cause insulin resistance (Figure 1) [29]. The central nervous system (CNS) plays an important role to balance the energy equation by regulating energy intake and expenditures, in the context of the homeostatic regulation of body weight [6]. Insulin and leptin are transported from the circulation into the brain [30]. Signaling of these hormones in the hypothalamus inhibits food intake and increases energy expenditure. It has been reported that the consumption of a HFD induces pro-inflammatory responses and recruitment of microglia and astrocytes in the hypothalamus [31]. These inflammatory changes also cause insulin and leptin resistance via blocking of their receptor signaling [32].

The innate immune system recognizes infected microorganisms through germline-encoded pattern recognition receptors (PRRs), such as Toll-like receptors (TLRs) and nucleotide-binding oligomerization domain (NOD)-like receptors (NLRs). These receptors interact with pathogen-associated molecular patterns (PAMPs), including lipopolysaccharide (LPS), peptidoglycan (PGN), bacterial DNA, and double stranded (ds)-viral RNA, which are essential for the survival of microorganisms [33]. PRRs also recognize endogenous damage-associated molecular patterns (DAMPs) derived from dead cells or tissue injury [33]. Low levels of DAMPs are beneficial during tissue repair to induce physiological immune responses and promote clearance [34]. However, recent studies have suggested that excessive amounts of DAMPs induce chronic low-grade inflammation in various tissues, including adipose tissue, islets and CNS [34]. These responses are mediated, at least in part, by the activation of PRRs. In addition, IL-1β has been implicated in various non-microbial pro-inflammatory diseases, including atherosclerosis, gout, and T2DM [35]. The secretion of IL-1β by inflammatory cells is largely dependent on multiprotein complexes termed inflammasomes, of which the hallmark activity is the activation of caspase-1 [36].

In this review, we will focus on the role of PRRs, including TLRs, NLRs and inflammasomes, in the induction of obesity-associated inflammation and highlight links to insulin resistance. We recently identified the Radioprotective 105 (RP105)/myeloid differentiation (MD)-1 complex as a key regulator of diet-induced chronic inflammation in adipose tissue, obesity and insulin resistance that appear to be independent of the TLR4-dependent pathway [37]. So, we will also overview our recent research regarding RP105/MD-1 and discuss potential mechanisms by which RP105/MD-1 is involved in chronic adipose tissue inflammation.

## 2. Toll-Like Receptors

The TLR family consists of at least 10 members in humans and 13 in mice. TLRs are type I transmembrane glycoproteins with cytoplasmic signaling domains and extracellular domains. The extracellular domains are composed of 18–25 tandem copies of leucine-rich repeat (LRR) motifs, which form the concave surface and ligand binding domains. The activation of TLR signaling pathways originates from a cytoplasmic Toll-IL-1 receptor (TIR) domain that associates with a different combination of TIR domains containing adaptor molecules [38]. Different TLRs utilize different adaptors to induce the production of pro-inflammatory cytokines [39]. Myeloid differentiation primary response gene 88 (MyD88) recruits different interleukin-1 receptor-associated kinase (IRAK) family proteins and activates TNF receptor-associated factor 6 (TRAF6) [40]. This complex activates TAK1 (TGF-activated kinase 1), leading to activation of nuclear factor-κB (NF-κB) and mitogen activated protein kinases (MAPKs) [39]. TIR-domain-containing adaptor-inducing interferon (TRIF) associates with receptor interacting protein 1 (RIP1) and TRAF6 [39]. The association of RIP1 and TRAF6 leads to activation of NF-κB and MAPKs to induce pro-inflammatory cytokines [39]. TRAF3 activation initiates the phosphorylation and activation of interferon regulatory factor 3 (IRF3), leading to IFN-γ production [40].

### 2.1. Toll-Like Receptor 4

TLR4 is widely expressed by hematopoietic and non-hematopoietic cells, and associated with a secreted MD-2 protein [41,42]. MD-2 recognizes LPS [43,44] and induces dimerization of heterodimeric TLR4/MD-2 complexes, leading to dimerization of cytoplasmic signal domains [41,42,44,45]. There is a body of evidence suggesting that TLR4 is an attractive candidate for linking innate immune responses to insulin resistance. First, TLR4 expression is increased in adipose tissue inflammatory macrophages in obesity [46,47]; Second, TLR4 knockout (KO) mice or mice with a loss-of-function mutation in the *TLR4* gene are protected from obesity-induced insulin resistance [46,48]; Third, hematopoietic cell-specific deletion of TLR4 ameliorates HFD-induced hepatic and adipose tissue insulin resistance [49]; Fourth, saturated fatty acids (FAs) released by adipocyte lipolysis activate the NF-κB pathway *in vitro* through TLR4 on macrophages [50]; Fifth, of interest, G-protein-coupled receptor 120 recognizes unsaturated omega-3 FAs such as docosahexaenoic acid and inhibits insulin resistance by suppressing TLR4-mediated macrophage activation [51].

It has been reported that FAs modulate TLR4-mediated signaling pathways and induce inflammatory gene expression [52]. Plasma free FA (FFA) levels are increased in obesity because hypertrophied adipocytes release FFA [53]. Saturated FAs, including lauric acid, palmitic acid and stearic acid, induce inflammatory responses through TLR4 [52,54,55]. Crystal structure analysis shows that MD-2 has a deep hydrophobic cavity, in which four FA chains of lipid A are all deeply confined [43,44,45]. Also, it has been reported that palmitate interacts with the TLR4/MD-2 complex [56,57]. Collectively, MD-2 may directly interact with saturated FAs and activate TLR4-mediated signaling pathways.

Since TLR4 is expressed in various cells, its function may be dependent on different types of cells and organs. In adipose tissue, it is proposed that interactions between adipocytes and macrophages through TLR4/MD-2 aggravates adipose tissue inflammation (Figure 1) [8]. FFAs derived from adipocytes would activate macrophages through direct or indirect interaction of the TLR4/MD-2 complex and induce TNF-α production [50,58]. Then, secreted TNF-α activates adipocytes and leads to production of monocyte chemotactic protein-1 (MCP-1)/CC chemokine ligand (CCL) 2 and chemokine (C-X-C motif) ligand 1 (CXCL1) to recruit macrophages into adipose tissue. In pancreas, cells themselves would be activated by FFAs through TLR4/MD-2 and produce CCL1 and MCP-1/CCL2, which recruit macrophages into the pancreas (Figure 1) [57]. Infiltrated M1-like macrophages are a source of pro-inflammatory cytokines that induce cell dysfunction [57].

However, other studies have suggested that FFAs do not directly activate TLR4-mediated signaling pathways. Erridge and Samani demonstrated that the effects of FFAs are influenced by the contamination of LPS in bovine serum albumin (BSA) used for solubilizing FFAs [59]. Another report showed that TLR4 signaling is not directly required for saturated or unsaturated fat-induced hepatic insulin resistance in mouse models [60]. Furthermore, plasma concentration of LPS is moderately increased by HFD and LPS is responsible for obesity and insulin resistance [61]. This metabolic endotoxemia could be due to increased intestinal permeability and enhanced LPS absorption by HFD [62]. In contrast, a recent paper demonstrated that FFA-induced inflammatory responses are TLR-dependent, but not due to the contamination of LPS [63]. Therefore, whether direct interactions between FFAs and the TLR4/MD-2 complex activate TLR4 signaling pathways remains controversial. Further studies are needed in order to understand the precise molecular mechanisms by which FFAs activate TLR4 signaling pathways.

Proteins including fetuin-A [64] and resistin are reported to be endogenous ligands for TLR4 and induce insulin resistance [65]. Fetuin-A, a liver secretory glycoprotein, is induced by FFA stimulation through the NF-κB pathway and acts as a carrier protein of FFA in the circulation [66]. Fetuin-A-deficient mice are protected from insulin resistance, indicating that it plays an essential role in disease [65]. Pal *et al.* [64] reported that fetuin-A directly interacts with TLR4, and a FFA-fetuin-A complex causes insulin resistance, suggesting that fetuin-A mediates the interaction of FFA with TLR4/MD-2. Benomar *et al.* [65] reported that resistin, an adipose-derived hormone, directly binds to TLR4 and induces insulin resistance.

**Figure 2:**
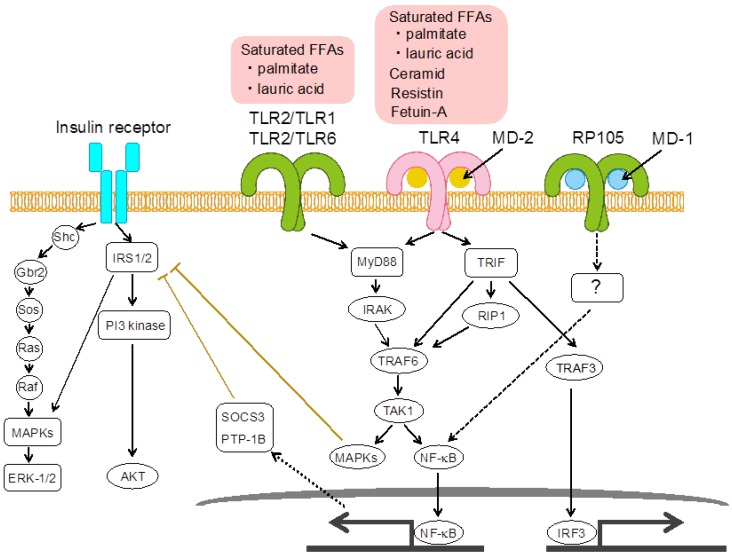
Insulin signaling pathways are inhibited by damage-associated molecular pattern (DAMP)-mediated, Toll-like receptor (TLR)-mediated pathways. TLR2/TLR1, TLR2/TLR6 and TLR4/MD-2 activate both nuclear factor-κB (NF-κB) and mitogen activated protein kinase (MAPK) pathways by recognition of various DAMPs. Suppressor of cytokine signaling (SOCS)-3, protein tyrosine phosphatase (PTP)-B1 and MAPKs such as c-Jun *N*-terminal kinase (JNK) 1 inhibit insulin receptor substrate (IRS) 1/2 phosphorylation of insulin receptors. Signaling via Radioprotective 105 (RP105)/myeloid differentiation protein-1 (MD-1) may activate the NF-κB pathway, but precise RP105-mediated mechanisms and the contribution to adipose tissue inflammation remain unclear.

Insulin receptors are expressed on adipocytes [67], skeletal myocytes [68], hepatocytes [69], and neurons in the hypothalamus [70]. Insulin signaling is transduced by two pathways (Figure 2) [71]. One pathway proceeds through insulin receptor substrate (IRS)-1/2 and depends on activation of phosphatidylinositol (PI) 3-kinase [71]. Another pathway proceeds through binding of tyrosine-phosphorylated IRS-1/2 or Shc with Grb2/Sos, leading via p21Ras and Raf-1 to activation of MAPK isoforms of extracellular signal–regulated kinase (ERK)-1 and -2. Phosphorylation sites of IRS have positive and negative effects on insulin signaling [72,73]. Also, TLR4 signaling through MyD88 is known to regulate insulin signaling (Figure 2) [13]. The serine residues of IRS are phosphorylated by MAPKs through the activation of TLR4 signaling [4]. In addition, the expression of the suppressor of cytokine signaling (SOCS)-3 and protein tyrosine phosphatase (PTP)-1B, which are considered to be major negative regulators of insulin signaling, is induced by ligation of TLR4 [74].

### 2.2. Radioprotective 105

RP105 is a homolog of TLR4 and associates with MD-1, a homolog of MD-2 [75]. RP105 as well as TLR4 contains 22 LRRs in their extracellular portions, while RP105 has only 11 amino acids in the intracellular portion and lacks a TIR domain [76,77]. In B cells, the interaction of anti-RP105 monoclonal antibody with RP105 transmits powerful activation signals independent of MyD88 by mediating the B-cell-specific signaling component, the Lyn/CD19/Vav complex [78,79]. In addition, RP105 KO mice as well as MD-1 KO mice show reduced LPS-dependent proliferation and CD86 up-regulation in B cells, indicating that the RP105/MD-1 complex functions as a complementary receptor, and augments TLR4/MD-2-mediated LPS responses [80,81]. In contrast, RP105- or MD-1-deficient macrophages or dendritic cells (DCs) are not impaired in TNF-α and IL-12 production in response to lipid A [82], suggesting that the RP105/MD-1 complex is not involved in LPS responses in myeloid cells. Divanovic and colleagues reported that RP105/MD-1 may negatively regulate TLR4/MD-2-mediated LPS responses in DCs [83]. However, precise roles of RP105/MD-1 in LPS responses remain elusive.

As analysis of RP105/MD-1 expression has been largely restricted to the immune system, we have examined whether RP105/MD-1 has a role in sensing an obesity-related endogenous ligand or whether this complex participates in immune responses leading to diet-induced adipose tissue inflammation and insulin resistance [37,84]. Of interest, the expression of RP105 mRNA is markedly increased by HFD in the stromal vascular fraction of epididymal white adipose tissue (eWAT) in wild-type (WT) mice. In humans, RP105 mRNA expression is also increased in the visceral adipose tissue of obese subjects relative to non-obese subjects. A majority of RP105-expressing cells in eWAT from WT mice are F4/80+ and CD11b/Mac-1+ ATMs. Some RP105-expressing cells in eWAT are B220+ and CD19+ B cells, but these populations are very small. HFD also increases levels of cell surface RP105/MD-1 expression on the M1 but not M2 ATMs and B cells in eWAT [37]. Using a co-culture system composed of 3T3-L1 adipocytes and macrophage cell lines, we have shown that RP105 and MD-1 mRNA expression are increased in macrophages in parallel with the up-regulation of TNF-α mRNA expression, although to a lesser extent the expression of TLR4 and MD-2 mRNA is observed in the contact co-culture system. These results suggest that RP105/MD-1 may play an important role in macrophage activation and adipose tissue inflammation. However, we could not exclude the possibility that RP105 in B cells and other cell types may involve in diet-induced adipose tissue inflammation. Of interest, RP105 KO and MD-1 KO mice have less HFD-induced obesity, adipose tissue inflammation, hepatic steatosis and insulin resistance compared to WT and TLR4 KO mice. These results clearly suggest that the induction of obesity-related inflammation and metabolic disorders by HFD requires or is dependent on the RP105/MD-1 pathway.

It remains unclear why knockout of only RP105 or MD-1 has such a dramatic effect in diet-induced inflammation and metabolic disorders. As is demonstrated, RP105/MD-1 links TLR4/MD-2 and TLR2 in B cell responses to microbial components [82]. Furthermore, RP105/MD-1 interacts with TLR2 and regulates macrophages responses to *Mycobacterium tuberculosis* [85]. Thus, RP105/MD-1 may be an accessory molecule for both TLR4/MD-2 and TLR2. Previous studies suggested that TLR2 is critical for diet-induced adiposity and metabolic disorders in a mouse model [86,87]. Therefore, RP105 or MD-1 KO mice may impair both TLR4- and TLR2-mediated promotion of adipose tissue inflammation and insulin resistance. This information may provide a possible explanation for the dramatic effects of RP105- or MD-1-deficiency on diet-induced inflammation and metabolic disorders.

Intriguingly, RP105/MD-1 does not recognize palmitic acid, since RP105-deficient or MD-1-deficient macrophages normally respond to the FA as well as WT macrophages [37]. Western blot analyses show that both TLR4 and RP105 pathways are involved in HFD-induced NF-κB activation in the eWAT, while c-Jun *N*-terminal kinase (JNK) protein is phosphorylated by TLR4 activation, but not RP105 activation in the eWAT [37]. Our results suggest that ligands and signaling pathways involving RP105/MD-1 do not completely overlap with those utilized by TLR4/MD-2. As RP105 does not have a TIR domain, identification of a signal transducer for RP105 is required to clarify a RP105-mediated signaling pathway. Further studies will determine the precise actions of RP105/MD-1 in adipose tissue, including an endogenous ligand and a signaling pathway (Figure 2). Furthermore, it will be exciting to examine if this complex has roles in human obesity, metabolic disorders and atherosclerosis.

### 2.3. Toll-Like Receptor 2

TLR2 recognizes a variety of microbial components such as bacterial lipopeptides, PGN and lipoteichoic acids, and forms a heterodimer with TLR1 or TLR6 to discriminate their ligands [88,89,90,91]. TLR2/TLR1 and TLR2/TLR6 complexes recognize a triacetylated lipopeptide and a diacetylated lipopeptide, respectively, suggesting that FA chains may be critical for the TLR2-dependent ligand discrimination [90]. Interestingly, a saturated FA lauric acid induces NF-κB activation through TLR2 dimerized with TLR1 or TLR6 (Figure 2) [47,92,93,94]. Another paper reported that palmitate stimulation in a myoblast cell line activates MAPK and NF-κB activation through TLR2-MyD88 signaling and these inflammatory responses lead to insulin resistance in muscle tissue (Figure 2) [93].

Previous *in vivo* studies have implicated TLR2 in the pathogenesis of obesity and metabolic disorders [86,87]. TLR2 KO mice are substantially protected from diet-induced adiposity, insulin resistance, hypercholesterolemia and hepatic steatosis [86,87]. In contrast to these reports, Carcilli *et al.* [95] showed that TLR2 KO mice in conventionalized conditions increase insulin resistance associated with alterations in the composition of the gut microbiota, which display an increase in the relative abundance of Firmicutes and Bacteroidetes and decreased relative abundance of Proteobacteria, compared to their controls. The insulin resistance in TLR2 KO mice could be explained by the increased LPS serum levels, which lead to TLR4 activation in the insulin target organs [95]. Although the gut microbiota has been not investigated in most of the previous TLR2 KO studies, the authors suggest that TLR2-deficiency associated with different environments can induce different phenotypes, probably induced by different microbiotas [95]. In addition, a reduction in Treg cells in eWAT from TLR2 KO mice may also contribute to the increased insulin resistance in these mice [95]. Intriguingly, another group reported that TLR2 KO mice exhibit mature-onset obesity by modulating food intake [96]. So, the role of TLR2 in obesity-induced metabolic disorders merits more study in different organs.

### 2.4. Toll-Like Receptor 5

TLR5 is highly expressed on intestinal lamina propria DCs (LPDCs) [97]. TLR5 recognizes flagellin monomers, the primary structural component of flagella, and triggers pro-inflammatory responses [98]. TLR5-stimulated LPDCs induce the differentiation of naive B cells into IgA^+^ plasma cells and trigger the differentiation of antigen-specific Th17 and Th1 cells [97]. Additionally, TLR5 signaling in LPDCs induces IL-23 production and activates innate lymphoid cells to produce IL-22 in response to IL-23 [99,100]. These cytokines are particularly important for anti-microbial immunity and tissue repair via the induction of anti-microbial peptides and proliferation and survival of epithelial cells [101]. Therefore, TLR5 signaling has a crucial role in the maintenance of gut homeostasis [102].

It has been known that microflora have an important role in the production of simple sugars, absorbable nutrients, and FAs from indigestible food substances by fermentation [103]. In addition, the microbiome from obese mice has increased capacity to harvest energy from the diet [103]. TLR5 KO mice exhibit hyperphagia and develop hyperlipidemia, insulin resistance and increased adiposity [104]. TLR5 KO mice differ from WT mice regarding intestinal microbiome composition [104]. Interestingly, the transfer of the gut microbiota from TLR5 KO mice to WT germ-free mice confers many features of metabolic syndrome in the recipients [104]. These results suggest that TLR5 signaling may prevent abnormal proliferation of microbiota and these microbiota may be responsible for hyperphagia and obesity in TLR5-deficeint mice [104].

## 3. Nucleotide-Binding Oligomerization Domain (NOD)-Like Receptors and Inflammasomes

NLRs play essential roles in innate immunity by detecting cytosolic PAMPs and DAMPs [105,106]. The typical NLR is composed of three distinct common domains: *C*-terminal LRR domain that is involved in recognition of PAMPs and DAMPs; a centrally located nucleotide-binding and oligomerization domain (NACHT) that enables activation of signaling complex via adenosine triphosphate (ATP) dependent oligomerization and is essential for activation of NLRs; and *N*-terminal caspase recruitment domain (CARD) and pyrin domain (PYD), which mediate homotypic protein-protein interactions for downstream signaling [107,108].

Inflammasomes are cytoplasmic multiprotein complexes that trigger the maturation of pro-inflammatory cytokines, such as IL-1β and IL-18, hence referred to as a caspase-1-activation platform [109]. Inflammasomes consist of a sensor NLR protein or ds-DNA sensor absent in melanoma 2 (AIM2), the inflammatory protease caspase-1 and/or adapter protein apoptosis-associated speck-like protein containing a CARD (ASC) containing PYD-and CARD-domain [110]. So far, five types of inflammasome have been identified: NLRP1, NLRP3, NLRP6, NOD-like receptor subfamily C (NLRC) 4, and AIM2 inflammasome. Each inflammasome recognizes microbial and endogenous ligands, and recruits pro-caspase-1 by either directly interacting through CARD domains or indirectly through ASC, resulting in the formation of active caspase-1 [111]. Activated caspase-1 subsequently cleaves pro-IL-1β and pro-IL-18 and induces their releases via a non-classical secretion pathway [112,113,114]. Mature IL-1β has been linked to many immune reactions, including the recruitment of inflammatory cells [115,116].

### 3.1. Nucleotide-Binding Oligomerization Domain (NOD)-Like Receptors

NOD1 and NOD2 are key members of NLR family and recognize bacterial PGN moieties as intracellular receptors. NOD1 recognizes d-glutanyl-meso-diaminopimelic acid derived from Gram-positive bacteria, whereas NOD2 recognizes muranyl dipeptide derived from both Gram-positive and Gram-negative bacteria [117,118,119]. NOD1 or NOD2 recognizes their ligands through the LRR domain and NACHT domains oligomerize, imitating the recruitment of receptor-interacting protein 2 (RIP2) through CARD-CARD interactions [120,121]. RIP2 activation recruits TAK1-binding proteins (TAB)/TAK1 complexes to mediate activation of the NF-κB pathway and the phosphorylation of MAPKs, such as JNK, ERK and p38 MAPK, through the upstream activation of MAPK kinases (MKKs) [122,123,124,125].

**Figure 3:**
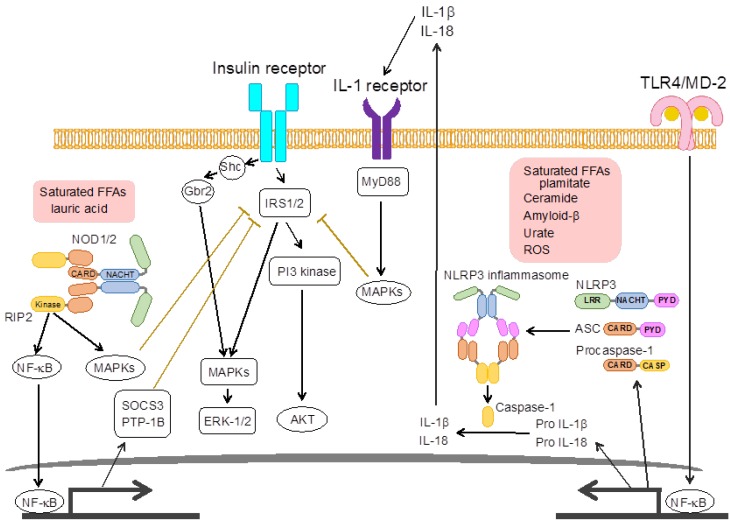
Signaling pathways used by nucleotide-binding oligomerization domain (NOD)1/2 and NOD-like receptor family, pyrin domain containing (NLRP) 3 inflammasomes for recognition of DAMPs and inhibition of insulin signaling. NOD1/2 are activated by FFAs such as lauric acid and oligomerized, which recruits receptor-interacting protein 2 (RIP2) and activates both NF-κB and MAPK pathways. SOCS-3, PTP-B1 and MAPKs such as JNK1 inhibit IRS1/2 phosphorylation of insulin receptor signaling. TLR4/MD-2-mediated NF-κB activation induces the expression of NLRP3 and precursors of IL-1β/IL-18. NLRP3 recognizes various DAMPs and is oligomerized, leading to the activation of caspase-1 and the production of mature IL-1β/IL-18. Secreted IL-1β activates MAPKs via IL-1 receptor (IL-1R) and inhibits insulin receptor signaling by serine phosphorylation of IRS1/2.

Accumulating evidence indicates that activation of NOD1 and NOD2 is associated with insulin resistance. Schertzer *et al.* [126] reported that NOD1/2 double KO mice are protected from HFD-induced inflammation, lipid accumulation, and insulin resistance in adipose tissue and liver. Administration of NOD1 ligand but not NOD2 ligand induces inflammatory reactions in adipose tissue, and impairs insulin signaling and glucose uptake in adipocytes and liver [126,127,128]. Lauric acid can activate the NF-κB pathway through NOD1/2, suggesting the saturated FAs may be an endogenous ligand for NOD1/2 (Figure 3) [129,130].

### 3.2. NOD-Like Receptor Family, Pyrin Domain Containing 3

NLRP3 is expressed in myeloid cells, including DCs, monocytes, macrophages, and neutrophils, while levels are up-regulated in response to PAMPs and DAMPs [131]. In general, NLRP3 signaling is regulated in two different steps. The first step involves a priming signal to induce expression of NLRP3 and pro-IL-1β, which is mediated by NF-κB activation through TLRs and IL-1 receptor (IL-1R). The second one is the activation of the NLRP3 inflammasome which results in caspase-1 activation and three major models are widely supported [132]: (1) extracellar ATP triggers P2X7-dependent pannexin-1 membrane pore formation, allowing extracellar NLRP3 agonist to access the cytosol and directly activate NLRP3 [133]; (2) The engulfment of crystalline and particulate substances, such as monosodium urate [134], silica [135], amyloid-β [136,137], and alum [135], lead to liposomal rupture, resulting in release of liposomal contents that activate the NLRP3 inflammasome [134,135,136,137]; (3) Reactive oxygen species (ROS), which are generated by cellular stress including bacterial infection, phagocytosis, and metabolic stress, activate the NLRP3 inflammasome [115]. On the other hand, a few molecules, such as amyloid-β, can induce both NLRP3 priming through TLR activation and NLRP3 inflammasome activation [136].

Accumulating evidence supports the idea that the NLRP3 inflammasome has a critical role in the development of adipose tissue inflammation and insulin resistance [35,138]. In adipose tissue, NLRP3 is localized in F4/80+ macrophages and leads to strong caspase-1 auto-activation [139]. Expression of NLRP3, ASC, and caspase-1 are up-regulated in adipose tissue from HFD-fed obese mice [139]. Consistent with caspase-1 activation, levels of the mature form of IL-1β are increased in adipose tissue from HFD-fed mice [140]. Then, secreted IL-1β activates NF-κB and MAPK pathways through the IL-1R and impairs insulin signaling by regulating the IRS-1 signaling pathway (Figure 3) [139,141]. In addition, NLRP3 KO and caspase-1 KO mice have improved HFD-induced insulin resistance and inflammation compared to WT mice [139,140,142]. Moreover, weight loss in obese patients with T2DM is associated with insulin sensitivity and the reduction of NLRP3 and IL-1β expression in adipose tissue [139]. Lee *et al.* [143] showed that levels of IL-1β maturation and caspase-1 cleavage are decreased in monocyte-derived macrophages from T2DM patients treated with metformin, an anti-diabetic drug. These reports indicate that NLRP3 inflammasome plays an impotent role in T2DM in humans as well as mice.

Non-alcoholic fatty liver disease (NAFLD) is considered to be the manifestation of metabolic disorders. A recent paper suggests that NLRP3- and NLRP6-dependent processing of IL-1β and IL-18 may have an important role in the pathogenesis of NAFLD as well as multiple aspects of metabolic syndrome via modulation of the gut microbiota [144]. In the gut of inflammasome-deficient mice, abnormal accumulation of bacterial products is observed in the portal circulation and the liver is exposed to the highest concentration of those bacterial products such as PAMPs [144].

Several molecules have been identified as DAMPs involved in NLRP3 activation in diet-induced obesity (Figure 3) [35]. Ceramide, composed of sphingosine and a FA, can induce the activation of caspase-1 and release of IL-1β in an NLRP3-dependent manner [139]. A saturated FFA palmitate also induces the activation of caspase-1 and production of mature IL-1β and these responses are inhibited by a ROS inhibitor [141]. Since ceramide and palmitate would be considered to be as endogenous TLR4 ligands and induce mitochondrial ROS production by transducing of TLR4 signaling to mitochondria in macrophages [145,146], ROS may be crucial intermediates for activation of NLRP3. In addition, islet amyloid polypeptide (IAPP), which is accumulated in islet of T2DM patients, is also an inducer of NLRP3 activation [137].

## 4. Concluding Remarks

As described herein, since the diverse cells express various kinds of PRRs, the complex effects of PRRs are integrated into obesity-induced chronic inflammation by interacting with endogenous ligands. In addition, the change of intestinal microflora and the increase in the serum level of exogenous ligands such as LPS are also involved in obesity-induced chronic inflammation, suggesting that both exogenous and endogenous ligands may trigger the inflammation. Our recent study demonstrates that the RP105/MD-1 complex could be a novel therapeutic target for obesity-associated metabolic disorders [37]. Further studies are required to clarify mechanisms by which PRRs recognize their ligands and induce the inflammation. Successful development of new therapeutics is likely but will require a much greater understanding of these ligand/receptor interactions.
